# 端粒与端粒酶研究在肺癌中的临床应用前景与挑战

**DOI:** 10.3779/j.issn.1009-3419.2020.102.45

**Published:** 2021-01-20

**Authors:** 森 韩, 旭 马, 健 方

**Affiliations:** 100142 北京，北京大学肿瘤医院暨北京市肿瘤防治研究所恶性肿瘤发病机制及转化研究教育部重点实验室 Key Laboratory of Carcinogenesis and Translational Research (Ministry of Education), Peking University Cancer Hospital & Institute, Beijing 100142, China

**Keywords:** 端粒, 端粒酶, 肺肿瘤, 端粒酶抑制剂, Telomere, Telomerase, Lung neoplasms, Telomerase inhibitor

## Abstract

肺癌是世界范围内发病率和死亡率较高的恶性肿瘤之一。端粒和端粒酶与肺癌的发生发展密切相关。虽然端粒酶可能不是导致细胞癌变的直接原因，但在维持端粒长度和肿瘤生长方面起到关键作用。包括肺癌在内的大部分肿瘤端粒长度缩短。端粒长度的变化与肺癌发生风险相关，并可能成为肺癌的治疗靶标和预测指标。针对端粒和端粒酶信号通路的靶向治疗药物正在探索中，以端粒酶抑制剂为代表的小分子药物有希望应用于肺癌的临床治疗中。但是，人们对于端粒和端粒酶的研究还远远不够，端粒长度维持的旁路作用机制可能是下一步需要深入研究的方向。

## Abstract (en)

Lung cancer is one of the malignant tumors with high incidence rate and high mortality worldwide. Telomere and telomerase are closely related to the occurrence and development of lung cancer. Although telomerase may not be the direct cause of carcinogenesis, it plays a key role in maintaining telomere length and tumor growth. The length of most tumors, including lung cancer, is shortened. The change of telomere length is related to the risk of lung cancer, and may become the therapeutic target and predictive index. Target drugs for telomere and telomerase signaling pathway are constantly being explored, and drugs represented by telomerase inhibitors are expected to be used in clinical treatment of lung cancer in the future. However, the research on telomere and telomerase is far from enough. The bypass mechanism of telomere length maintenance may be the direction of further research.

从20世纪30年代开始，人们逐渐了解染色体上的一段特殊结构——端粒（telomere）。端粒位于真核细胞染色体的末端，与端粒结合蛋白一起构成特殊的“帽状”结构，保持染色体的完整性和控制细胞分裂周期。其实质为一小段DNA——蛋白质的复合体。根据DNA复制的机制及特点，每次染色体复制后，延伸链上的DNA末端必有一小段无法被复制。所以，细胞每分裂一次，端粒缩短30 bp-200 bp，当其缩短到2 kb-4 kb的特定临界值时，细胞将会进入静默期或者启动凋亡机制。因此，端粒被推测与细胞老化有明显的关系^[1]^。但是，人体的部分细胞，例如精原母细胞、肿瘤细胞等，含有端粒酶，能在DNA末端接上新的端粒片段，使其不会随着分裂次数增加而缩短，从而实现细胞的无限复制。端粒酶也被认为与肿瘤有着密切的关系^[2]^。2009年诺贝尔生理学或医学奖授予在端粒和端粒酶研究领域有突出贡献的伊丽莎白·布莱克本等3位科学家，以表彰他们发现了端粒和端粒酶保护染色体的机制。从此，端粒与端粒酶的研究在肿瘤领域受到越来越多的关注，尤其是阻断端粒延长机制以及端粒酶抑制剂等可能成为潜在的抗肿瘤治疗新靶点^[3]^。

肺癌是世界范围内发病率和死亡率最高的恶性肿瘤，据统计每年有超过一百万人死于肺癌^[4]^。肺癌分为小细胞肺癌（small cell lung cancer, SCLC）和非小细胞肺癌（non-small cell lung cancer, NSCLC），二者在临床病理特征及预后方面存在很大差异。总体来说，肺癌的5年生存率只有18%左右^[5]^。虽然随着近年来抗肿瘤治疗手段的提高，靶向药物和免疫治疗极大地改善了进展期肺癌患者的预后，但是靶向和免疫治疗的耐药仍不可避免^[6, 7]^。为了能够更好地控制肿瘤，人们在端粒和端粒酶的研究方面也做出积极探索。不仅如此，端粒及端粒酶在肿瘤及正常组织中的差异，使其可能成为天然有效的抗肿瘤治疗靶标和预测指标，近年来也有许多相关报道。因此，本文将对端粒和端粒酶研究在肺癌领域中的临床应用进展进行阐述，并提出该研究领域的发展前景和面临的挑战。

## 1 端粒与端粒酶

端粒是真核细胞线性染色体的末端结构，在细胞复制过程中起保护作用，避免DNA受到损伤，并且像帽子一样有效防止染色体间末端重组、融合和染色体退化。端粒的DNA序列是高度保守的，所有哺乳动物的端粒是由“d-TTAGGG”重复序列组成，并且包含六种蛋白质而形成的特定“shelterin”复合体，维持染色体和基因组的稳定性^[8]^。正常体细胞受精后，端粒随着每次细胞分裂和染色体复制而逐渐缩短，这导致一旦端粒缩短达到特定的长度（称为“Hayflick极限”），细胞就会进入静止期，从而停止分裂，或者启动细胞凋亡机制。由于大部分体细胞中缺乏端粒延长的机制，所以该过程很大程度上参与了正常细胞的衰老过程。具有增殖功能的干细胞和癌细胞可以通过一种功能性核糖核蛋白酶复合物，即端粒酶，来解决端粒缩短问题。

端粒酶是一种由RNA和蛋白质组成的核糖核蛋白体复合体。端粒酶属于一种逆转录酶，与端粒（长度）的调控机制密切相关。人类端粒酶的组成包括：端粒酶RNA（human telomerase RNA component, hTR）、端粒酶活性催化单位（human telomerase reverse transcriptase, hTERT）以及端粒酶结合蛋白。它以自身的RNA作为端粒DNA复制的模板，合成出富含脱氧单磷酸鸟苷（G）的DNA序列后，添加到染色体的末端并与端粒蛋白质结合，从而稳定了染色体的结构。在正常人体细胞中，端粒酶的活性受到严格调控，只有在癌细胞、干细胞和生殖细胞中才能检测到具有活性的端粒酶。

## 2 端粒、端粒酶与肿瘤

相对于正常体细胞，肿瘤细胞的端粒一般较短。研究^[9, 10]^证实绝大多数膀胱癌的浸润前期、头颈癌、结肠癌、食管癌和口腔癌端粒长度非常短，而大多数肿瘤细胞通过端粒酶的激活维持端粒在一个稳定的长度，确保细胞的快速增殖及永生化。一个有趣的现象是：尽管在85%-90%的肿瘤中都能够检测到端粒酶的活性，但端粒酶并不是导致细胞癌变的直接原因，而是在肿瘤生长和增殖过程中具有决定性的作用。因此，端粒酶的激活发生在癌变之后（当第一个肿瘤细胞已经产生），端粒酶使得这个肿瘤细胞变得不朽，并能够无限分裂增殖。有时，端粒酶也是可以抑制肿瘤的。当端粒酶进入非活性状态，则会减少细胞分裂的数量，这成为人们利用端粒酶抑制剂进行抗肿瘤的机制。

## 3 端粒、端粒酶与肺癌

### 3.1 端粒长度与肺癌的发生风险

Jang等^[11]^研究端粒长度与肺癌发生风险之间的关系，结果显示端粒长度的缩短与肺癌发生风险升高相关。该研究通过定量聚合酶链反应测量了243例肺癌患者和243名健康对照者的外周血淋巴细胞相对端粒长度，这些患者的年龄、性别和吸烟状况均匹配。结果提示肺癌患者的端粒长度明显短于健康对照组（1.59 *vs* 2.16, *P* < 0.000, 1）。将端粒长度的中位数作为临界点时，端粒短的个体患肺癌的风险明显高于端粒长的个体（OR=3.15, 95%CI: 2.12-4.67, *P* < 0.000, 1）。当按肺癌组织亚型进行分类时，端粒长度缩短对肺癌发生风险影响最大的是SCLC，这一影响比鳞状细胞癌和腺癌均明显（*P*=0.001，均一性检验）。因此表明端粒的缩短可能是肺癌的危险因素，或可作为肺癌易感性的预测指标^[11]^。

但是，另一项针对重度吸烟人群的前瞻性观察研究^[12]^却得出不同的结论，即端粒长度增加的个体肺癌发生风险增加。该研究是一项包含709例肺癌患者和1, 313例健康个体的巢式病例对照研究，在端粒长度最长的人群中（采用三分位数），总体肺癌和肺腺癌的发生风险均升高，并且与肺腺癌的关联最强。一项针对26, 540名新加坡华人的健康随访^[13]^也得出了相似的结论。该研究测定所有受试者外周血白细胞的相对端粒长度，经过12年的随访，有654名观察对象罹患肺癌，其中包括288例腺癌、113例鳞状细胞癌和253种其他/未知组织学类型的肺癌。使用*Cox*风险回归模型来评估肺癌发生的风险，结果显示端粒长度前20%者与后20%者相比，肺腺癌的发生风险升高（HR=2.84, 95%CI: 1.94-4.14, *P* < 0.000, 1），而端粒长度与肺鳞癌和其他病理类型的发生风险无明显相关性^[13]^。

基于上述两种不同的观点，理论上分别有相应的解释：①端粒长度的缩短，使得细胞的基因不稳定性增加，因此容易发生基因突变等异常情况，使得肺癌的肿瘤发生风险增加；②端粒长度的延长，使得细胞寿命延长，获得了更多分裂的机会，而在更长的时间和更多的分裂机会下，发生基因突变的几率会升高。如果发生了肺癌肿瘤驱动基因的改变，那么发生肺癌的风险自然就会增加^[14]^。因此，两种截然相反的结果，都有其合理的理论解释。为了解答这一问题，对于端粒长度与肺癌发生风险的研究，尤其是机制方面的研究还有待进一步完善。

### 3.2 端粒长度与肺癌患者的预后

有研究^[15-17]^显示外周血白细胞中端粒长度与癌症患者的死亡风险相关。虽然不同研究得到的结果并不完全一致，但总体来说端粒长度的缩短提示肺癌患者的预后较差。一项针对13项非血液系统恶性肿瘤研究的荟萃分析^[18]^发现，外周血端粒长度缩短与癌症患者死亡风险增加相关。Weischer等^[15]^通过对丹麦人群的研究发现，诊断为肺癌之前检测到端粒长度缩短与肺癌患者死亡率增加有关。Kim等^[19]^研究发现，在早期NSCLC患者确诊时测得的端粒长度过长与根治性肺癌切除术后复发风险增加相关。Jeon等^[20]^为了明确端粒缩短对早期NSCLC患者生存的影响，通过定量聚合酶链反应在164例手术切除的NSCLC患者中测量了肿瘤组织中的相对端粒长度，并且分析端粒长度与总生存期（overall survival, OS）和无病生存期（disease free survival, DFS）之间的关联。根据端粒长度的四分位数分类，端粒长度最短的患者与端粒长度第2-第4个四分位数的患者相比，OS和DFS明显缩短（其中OS的调整后HR=2.67，95%CI：1.50-4.75，*P*=0.001；DFS调整后的HR=1.92，95%CI：1.17-3.14，*P*=0.01）。在鳞状细胞癌中，端粒长度与预后的关联比腺癌更为明显。因此，该研究的结论是手术切除的早期NSCLC患者的肿瘤组织端粒长度是肺癌患者独立的预后因素。

一项针对吸烟人群的大规模前瞻性研究^[21]^显示，端粒长度缩短与SCLC的死亡率增加密切相关，尤其是Ⅲ期/Ⅳ期的SCLC患者，端粒长度缩短者与较长者（采用三分位数）相比，死亡风险增加3.32倍（95%CI: 1.78-6.21）。但是端粒长度与肺癌其他病理类型的死亡风险无明显相关性。以上是基于788例肺癌患者，在确诊之前平均6年的时间里进行的端粒长度测定，并且随访8年时间而得出的结论。

### 3.3 端粒酶与肺癌及肺癌驱动基因

Dobija-Kubica等^[22]^报道了端粒酶活性与NSCLC的病理类型及其预后的关系。该研究检测了47例肺癌组织的端粒酶活性，并与30例肺癌旁的正常肺组织进行对比，结果发现肺癌组织中的端粒酶活性显著高于癌旁组织，并且分化越差的肺癌中端粒酶活性越高（*P*=0.008）。但是，端粒酶活性与病理类型和患者的2年生存率均无明显相关性^[22]^。

Xing等^[23]^通过对828例受试者（410例NSCLC，418名健康对照）进行*TERT*基因多态性检测发现，在rs2736100基因位点携带G的人群发生NSCLC的风险显著增高（TG+GG *vs* TT, OR=1.68, 95%CI: 1.28-2.07）。在rs2736098基因位点携带A的人群发生NSCLC的风险显著增高（GA+AA *vs* GG, OR=1.52, 95%CI: 1.19-1.93）。

表皮生长因子受体（epidermal growth factor receptor, *EGFR*）基因是NSCLC中最重要的肿瘤驱动基因。越来越多的研究^[24-26]^认识到EGFR信号通路可能与端粒酶的活性有关，*EGFR*可以通过增加TERT的转录，从而增强端粒酶的活性。Yuan等^[27]^研究*TERT*基因启动子区域基因多态性与肺癌的关系，结果发现在*EGFR*基因野生型的NSCLC中，rs2853669 T/C等位基因与TERT的低表达显著相关（与C/C和T/T相比，*P* < 0.001）；而在*EGFR*突变人群中无明显相关性。rs2853669 T/C等位基因与端粒长度缩短及肺癌患者的预后也存在明显相关性。Wei等^[28]^研究发现*TERT*基因位点rs2736100-C等位基因与*EGFR*基因突变呈显著相关性，而EGFR野生型无关。

### 3.4 端粒和端粒酶研究在肺癌治疗中的应用前景

肺癌是一种个性化比较丰富的肿瘤，临床上用药有效率不高，继续通过化疗、放疗、靶向治疗甚至是免疫治疗有效，肿瘤的耐药也不可避免，这一直困扰着肺癌临床治疗。虽然目前在端粒和端粒酶的相关领域内尚无一种药物可以成功用于临床抗肿瘤治疗，但人们正在从不同的角度积极进行靶向治疗药物的研究和探索，例如：端粒结构的类似物和端粒酶抑制剂等。

#### 3.4.1 鸟氨酸四联体稳定剂

正常情况下，端粒会形成鸟氨酸四联体（G-quadruples complex, G4）的二级结构，从而维持结构稳定和发挥正常的生理功能^[29]^。针对端粒的这一结构特点，一些小分子的靶向药物被设计出来，例如BRACO19、RHPS4、Telomestain等^[30]^。希望以此来阻断端粒与端粒酶的结合，从而阻止端粒的延长、抑制其对染色体的保护功能，达到抗肿瘤的作用。

#### 3.4.2 端粒末端单链核苷酸类似物

端粒3'末端单链核苷酸类似物（oligonucleotides mimicking the 3'overhang of telomere sequences, T-oligo）通过模拟端粒末端突出的单链，被细胞的防御机制识别为危机长度的端粒，启动类似DNA损伤的反应，进入细胞凋亡途径^[31]^。同时，T-oligo还可以阻断端粒与端粒酶的结合。总体来说，针对端粒的小分子靶向药物初步显示出较好的抗肿瘤效果，但是因其不良反应较严重，即对于增殖较快的正常细胞和干细胞产生损害，所以目前还不能进入临床应用阶段。另外，针对端粒的小分子靶向药物并不依赖端粒酶，所以对于端粒酶阴性的肿瘤也可以发挥抑制作用^[32]^。

#### 3.4.3 针对人类端粒酶逆转录酶模板RNA的反义寡核苷酸

针对人类端粒酶逆转录酶模板RNA的反义寡核苷酸属于端粒酶抑制剂，通过结合端粒酶RNA-蛋白复合体中的RNA部分，发挥抑制端粒酶功能的作用，例如端粒酶抑制剂Imetelstat（GRN163L）。一项旨在探讨Imetelstat在进展期NSCLC维持治疗中作用的Ⅱ期临床研究^[33]^显示，端粒长度缩短的患者可以从Imetelstat的维持治疗中得到生存获益，但是对整体患者而言并不能提高NSCLC的无进展生存率，反而增加3度以上骨髓抑制的不良反应。

#### 3.4.4 hTERT抑制剂

hTERT是端粒酶复合物的活性催化亚基，因此抑制hTERT也属于端粒酶抑制剂的范畴，例如BIBR1532。临床前研究^[34, 35]^显示BIBR1532可以有效地抑制多种肿瘤细胞端粒酶的活性，缩短端粒并最终导致肿瘤细胞的增殖阻滞和凋亡。还有研究^[36]^还显示BIBR1532能够增加NSCLC患者的放射治疗敏感性，提示未来其有可能成为一种放疗增敏剂。

#### 3.4.5 针对hTERT的免疫治疗

端粒酶中的部分肽段结构能够作为端粒酶相关抗原（telomerase-associated antigens, TAAs）活化CD4^+^及CD8^+^ T细胞，另一方面可以促进hTERT特异性细胞毒性T淋巴细胞的激活。这些特点使得TAAs具有产生强大的免疫杀伤反应的潜能，从而介导特异性的免疫杀伤效应。例如GV1001，在包括黑色素瘤、进展期NSCLC及肝癌等多项Ⅰ期/Ⅱ期临床试验中取得了较好的疗效以及安全性，但目前尚无成功的Ⅲ期临床试验，因此也未临床应用于肺癌治疗^[10]^。

#### 3.4.6 靶向结合端粒酶的小RNA

小RNA（microRNA, miRNA）作为一种内源性的非编码RNA，可以在转录后水平进行基因表达的调控，即与信使RNA（message RNA, mRNA）靶向结合，抑制mRNA的翻译过程或者促进mRNA的裂解消亡。因此一些miRNA可以影响端粒酶基因的表达，从而影响端粒酶的合成代谢及功能。有研究^[37, 38]^显示miR-138、miR-128、miR-1182、miR-133a、miR-342、miR-491和miR-541等小RNA都可以与hTERT的mRNA结合，负向调节hTERT的表达，因此这些小RNA未来有可能作为肿瘤抑制性的RNA，成为肺癌治疗的新靶点。

## 4 端粒和端粒酶研究面临的问题与挑战

### 4.1 端粒长度测定的局限性

通过外周血标本进行端粒长度的检测是简便易行的方法，因为获得血液标本相比肺组织标本具有更少的侵入性，容易获得，并且肺癌的血液标志物对筛查易感人群具有重要意义。但是，血液中端粒长度的缩短也与常见的年龄相关疾病有关，例如中心型肥胖、睡眠不足和高血压等^[39]^。另外，肿瘤治疗相关的混杂因素、端粒测定方法的统一标准等问题，使得端粒长度的结果判定存在很大的局限性。

### 4.2 端粒长度维持机制的旁路激活的问题

虽然肺癌中端粒长度的维持主要依靠端粒酶的作用，即端粒酶是维持端粒长度、促进细胞不断分裂增殖的主要因素，但是有研究^[40]^发现，还存在一些端粒酶以外的旁路机制，即不依赖端粒酶也可以维持肿瘤端粒的长度稳定。这使得单一的端粒酶抑制剂在抗肿瘤治疗方面的作用大大削弱。因此，下一步可能需要深入研究端粒长度维持的旁路激活机制，并研究研发端粒酶抑制剂与其他药物的联合应用，为开发新的抗肿瘤治疗药物提供理论依据。

### 4.3 针对端粒和端粒酶的靶向药物治疗肺癌面临的挑战

虽然近年来靶向端粒/端粒酶的抗肿瘤治疗取得了许多进展，研发出包括鸟氨酸四联体稳定剂、端粒末端单链核苷酸类似物、针对人类端粒酶逆转录酶模板RNA的反义寡核苷酸、hTERT抑制剂等多种药物，有的已进入临床试验阶段，但是仍然存在很多问题。例如：端粒酶抑制剂Imetelstat在进展期NSCLC维持治疗中使患者得到生存获益，但是并未提高NSCLC的无进展生存率，反而增加3度以上骨髓抑制的不良反应^[33]^，使其在未能成功应用于临床。端粒酶抑制剂存在局限性的主要原因为：由于正常机体内的干细胞、生殖细胞及增生较快的正常细胞也表现出端粒酶活性，所有靶向端粒酶的治疗很可能导致对正常组织的损害，从而表现出各种近期和远期的不良反应，很难在取得抗肿瘤疗效的同时避免正常组织及器官的损伤。

另外，肺癌是一种异质性很强的恶性肿瘤，不同的病理亚型、不同的分子分型对于药物的治疗反应相差很大。同样地，肺癌中有的端粒酶活性高，并且端粒酶在肿瘤进展中发挥着重要作用，那么这部分肺癌患者对于端粒酶抑制剂可能有较好的疗效。而那些端粒酶活性不高的肺癌，或者肿瘤进展不依赖端粒酶，或者存在端粒长度维持机制的旁路激活，可能对于端粒酶抑制剂的疗效就差。因此，端粒/端粒酶抑制剂要想在临床应用上取得重大进展，很可能也要寻求个体化的治疗方案，找到一些更精准的“驱动基因”或者“肿瘤靶点”。

## 5 小结

端粒和端粒酶与细胞衰老和肿瘤密切相关。虽然端粒酶可能并不是导致细胞癌变的直接原因，但是在维持端粒长度和肿瘤生长方面却发挥着重要作用。包括肺癌在内的大部分肿瘤端粒长度缩短。端粒长度的变化与肺癌发生风险相关，并可能作为肺癌，尤其是SCLC患者的预后指标。针对端粒和端粒酶作用机制的抗肿瘤靶向治疗正在不断探索中，其中以端粒酶抑制剂最具潜在治疗价值。但是，人们对于端粒和端粒酶在肺癌发生发展过程中的作用机制认识还远远不够，例如端粒长度维持的旁路激活机制等。因此，下一步需要继续深入研究端粒和端粒酶的作用机制，期待能够早日研发出应用于肺癌治疗的靶向药物。
